# Ultrasound-guided brachial plexus nerve block in a patient with a left palmar schwannoma: A case report

**DOI:** 10.1097/MD.0000000000033440

**Published:** 2023-03-31

**Authors:** Yan Qu, Haomin Yang, Lichun Wei, Guoning Su

**Affiliations:** a Kunming Medical University, Kunming, Yunnan, P. R. China; b Department of Anesthesiology, Affiliated Hospital of Yunnan University, Kunming, Yunnan, P. R. China; c Department of Anesthesiology, Infectious Disease Hospital of Lijiang City, Lijiang, Yunnan, P. R. China.

**Keywords:** brachial plexus nerve block, schwannoma

## Abstract

Ultrasound-guided brachial plexus block is a common anesthetic procedure used in upper extremity surgery. However, it may not be a suitable option for some patients.

**Patient concerns:**

A 17-year-old woman with the left palmar schwannoma scheduled for surgical treatment received ultrasound-guided brachial plexus block. The anesthesia modalities of the disease were discussed.

**Diagnoses:**

Based on the patient’s complaints and clinical appearance, provisional diagnosis of neurofibroma was considered.

**Interventions:**

In this case, we present a case of ultrasound-guided axillary brachial plexus block used for upper extremity surgery in this patient. It was not easily and painlessly reduced in the surgery, although the visual analogue scale score was 0 and no motor movements of the left arm and palm were observed. The pain was relieved by intravenous injection of 50 mcg remifentanil.

**Outcomes:**

Immunohistochemically labeled pathological examination confirmed the mass to be a schwannoma. There was no need to apply additional analgesia after surgery, although the patient felt numbness in the left thumb for 3 days follow up.

**Lessons:**

Even if there is painless when skin-cutting after implementation of brachial plexus block, the patient is painful when pulls the nerve around the tumor during excision. It is necessary to give an analgesic drug or anesthetize a single terminal nerve as a supplement for brachial plexus block in patients with schwannoma.

## 1. Introduction

Schwannomas are histologically benign tumors with a very low risk of spontaneous malignant transformation.^[1]^ Ultrasound for peripheral nerve localization and ultrasound-guided regional anesthesia are becoming increasingly popular. That increased interest also implies the necessity of choosing the best type of regional anesthesia. The brachial plexus nerve block with ultrasound guidance is a well-known type of regional anesthesia used in upper extremity surgeries. However, a few studies in the medical literature have reported on the use and the efficiency of the ultrasound-guided brachial plexus block with peripheral schwannoma for patients. This article presents detailed information about our experience using the ultrasound-guided axillary brachial plexus block technique for a case of peripheral schwannoma incision that was not easily and painlessly reduced.

## 2. Case presentation

A 17-year-old female patient with a left palmar mess beside the thenar muscle presented to the hospital for excision of palmar mass. It was painful when pressed on the mess upon examination. The ultrasound examination was performed and revealed the mess measuring approximately 1 cm in diameter, which demonstrated a diagnosis consistent with neurofibroma.

The brachial plexus can be identified surrounding the artery during the left axillary nerve block. A linear transducer was placed at the target area, and the anatomy of the region was reviewed. A 50-mm, 22-gauge needle was advanced under ultrasonographic guidance until the brachial plexus nerves were located around the axillary artery. After negative aspiration, 20 mL injections (1% lidocaine and 0.5% ropivacaine) was given and spared. There is no feeling of paresthesia during anesthesia. Ten minutes after the injection, the visual analogue scale score was 0 and no motor movements of the left arm and palm were observed.

**Figure 1.**
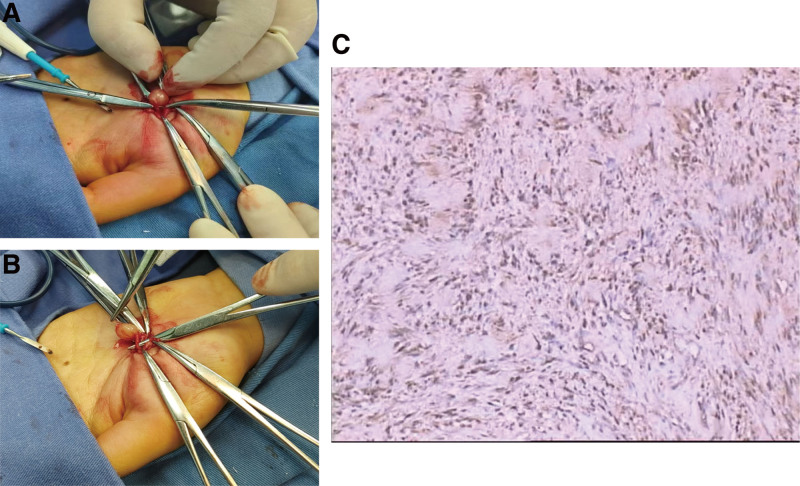
(A and B) Intraoperative photograph showing the schwannoma in the left palm prior to gross-total resection. (C) On immunohistochemical examination, tumor cells are positive for S-100 protein.

The 1.5 cm longitudinal incision on the surface of the mass was easily reduced without any pain. The mass was covered with a bundle of tiny nerve sheath as an envelope and clearly demarcated from the surrounding tissue (Fig. 1A and B). During the process of separation and stretching of the tiny nerve along nerve sheath of the mass, the patient experienced significant pain. The pain was relieved by intravenous injection of 50 mcg remifentanil. Immunohistochemically labeled pathological examination confirmed the mass to be a schwannoma. The tumor cells showed diffuse expression of S-100 protein (Fig. 1 C). There was no need to apply additional analgesia after this procedure, although the patient felt numbness in the left thumb for 3 days.

## 3. Discussion

Benign nerve sheath tumors include the schwannoma were named collectively as “neurofibromas” from their first description until this century.^[2,3]^ 29% of benign nerve sheath tumors in the upper extremities were schwannomas. Of these, 17% were involved the median nerve; 25%, the ulnar nerve; and 22%, the radial nerve.^[4,5]^ The schwannoma arises from Schwann cells like the neurofibroma.^[6]^ Symptoms of pain on palpation are characteristic of peripheral schwannomas, as noted in this case. It could be moved laterally, but not longitudinally in the direction in which the tumor reached. Microsurgical techniques are recommended for treatment of peripheral schwannomas. Percussion over the mass caused paresthesia’s in the distribution of the involved nerve in most patients with schwannomas. Clinical and pathological findings in patients of brachial plexus neurinoma reported that half of them still felt numbness and pain within 2 weeks after surgery.^[7]^

From the techniques for finding paresthesia described by Winnie in the mid- twentieth century, to the popularization of neurostimulators and ultrasounds, both anesthesiologists and their patients have benefited from technological advances. For using these techniques to enhance the quality of regional anesthesia can improve the accuracy and efficiency of nerve block and avoid the occurrence of related complications like nerves and blood vessels damage. Brachial plexus block at the level of the cords provides excellent anesthesia for procedures at or distal to the elbow. But for this patient brachial plexus block was incomplete. It is necessary to give an analgesic drug or anesthetize a single terminal nerve as a supplement when surgeons stretched the schwannoma although the patient’s visual analogue scale score was 0 and her left arm and palm could not be moved anymore.

For this patient, the target nerves are median nerve and palmar digital branches. The median nerve is derived from the lateral and medial cords of the brachial plexus. At the level of the proximal wrist flexion crease, it lies directly behind the palmaris longus tendon in the carpal tunnel. It enters the arm and runs just medial to the brachial artery. Just distal to this point, it gives off numerous motor branches to the wrist and finger flexors and follows the interosseous membrane to the wrist. Depending on visualized spread relative to the median nerve, we believed that 20 mL local anesthetics may be employed for intraoperative analgesia. While palmar digital branches as terminal nerves are crucial for patient with schwannoma in upper extremity. Terminal nerves may be anesthetized anywhere along their course, but the elbow and the wrist are the 2 most favored sites. Further study is warranted to determine a more applicable anesthetic management for such patients.

## Author contributions

**Data curation:** Haomin Yang.

**Resources:** Haomin Yang, Lichun Wei.

**Supervision:** Guoning Su.

**Visualization:** Yan Qu.

**Writing – original draft:** Yan Qu, Lichun Wei.

**Writing – review & editing:** Yan Qu.
